# Root Exudates Mediate the Production of Reactive Oxygen Species in Rhizosphere Soil: Formation Mechanisms and Ecological Effects

**DOI:** 10.3390/plants14091395

**Published:** 2025-05-06

**Authors:** Xuqin Wang, Yalei Liu, Xiaoyan Tian, Juan Guo, Yaning Luan, Dengzhi Wang

**Affiliations:** 1 Ordos Branch Station, Inner Mongolia Autonomous Region Environmental Monitoring General Station, Ordos 017000, China; wangxuqin81@163.com (X.W.); guojuan9540@163.com (J.G.); 2 The Key Laboratory for Silviculture and Conservation of Ministry of Education, College of Forestry, Beijing Forestry University, Beijing 100083, China; yaleiliu0218@bjfu.edu.cn (Y.L.); luanyaning@bjfu.edu.cn (Y.L.); 3 Department of Chemical Engineering, Ordos Vocational College, Ordos 017000, China; tianxiaoyan66@163.com

**Keywords:** reactive oxygen species (ROS), rhizosphere, Fenton reaction, plant response, decomposition and mineralization of organic matter, pollutant degradation

## Abstract

Reactive oxygen species (ROS), as redox messengers, play an important role in regulating plant growth, sensing biotic and abiotic stresses, and integrating different environmental signals. As the microenvironment of the interaction between root, soil and microorganism, the rhizosphere is the hotspot of ROS production and action. Root exudates are an important medium for communication between roots and the soil environment, and they have a significant regulatory effect on the production of ROS in the rhizosphere. At the same time, the formation of rhizosphere ROS is determined by the coupling of various biotic and abiotic factors, and it is also affected by environmental stresses such as temperature, humidity, and disease. This review summarizes how root exudates affect plant growth and induce plant defense mechanisms by regulating the generation and distribution of ROS. It also discusses the role of ROS in promoting the decomposition of soil organic matter, nutrient cycling, and pollutant degradation and transformation. In-depth study of the regulation mechanism of root exudates on ROS not only helps to reveal the molecular mechanism of plant adaptation to environmental stress but also provides theoretical support and practical guidance for sustainable agricultural development and ecological environment protection.

## 1. Introduction

Reactive oxygen species (ROS) are natural by-products of aerobic metabolic processes in organisms, including superoxide anion radicals (O_2_^•−^), hydroxyl radicals (^•^OH), hydrogen peroxide (H_2_O_2_) and other types, which have higher chemical reactivity than oxygen [1]. ROS are not only involved in the regulation of plant growth and development but also play a key role as signal molecules in mediating plant responses to biotic and abiotic stresses [2,3]. The production of ROS is the result of redox reactions involving molecular oxygen through the electron transfer reaction or high-energy exposure [2,4].

ROS are produced independently in most cell compartments, so their levels are strictly controlled to prevent unintended cell oxidation. The traditional view is that ROS are mainly potentially toxic to plants, so early research focused on the scavenging mechanism of ROS and their potential hazards [5,6,7]. However, with the deepening of research, more attention has been focused on the role of ROS as signal molecules. For example, cell homeostasis during the normal growth and development of plants is characterized by a baseline level of ROS, which depends on the developmental stage, circadian rhythm, environmental and physiological conditions of plants, as well as the interaction with the root and leaf microbiota. Different biotic and abiotic stresses may disrupt this homeostasis, uncoupling metabolic pathways and leading to the accumulation of ROS in different cell compartments [8,9,10].

In the deep rhizosphere, the absence of photochemical processes such as UV radiation has led to a greater research focus on ROS generated via light-driven reactions (e.g., in plant leaves and surface soils), resulting in relatively limited studies on ROS production mechanisms in aphotic environments. Since the root exudates in the rhizosphere soil can input abundant carbon sources and energy to attract and promote microbial activities, the rhizosphere soil is considered to be a microbial hotspot for biogeochemical processes in the soil [11,12]. There are abundant root exudates, enzyme activities, microbial activities and a unique pH at the rhizosphere interface, which participate in and affect the interface electron transfer and redox reactions. These reactions may induce the production and accumulation of ROS. For example, dark production of ROS can be induced by the exposure of extracellular electrons (generated by anaerobic microbial respiration) to molecular oxygen [13]. ROS can also be generated through electron donation from reduced iron minerals (Fe(II)-bearing clay minerals, pyrite) and natural organic matter (humic substances) under redox-oscillating conditions [14]. ROS production was observed in the rice rhizosphere, and the distribution of ROS showed a clear radial gradient pattern from the root surface to the surrounding environment. The production of extracellular ROS was triggered by the interaction between the oxygen released by the root system and the extracellular electrons released by the microbial respiration. Iron minerals and organic matter might play a key role in the storage and transfer of electrons [15]. The generated ROS can affect the degradation and transformation of soil pollutants, nutrient cycling and carbon accumulation in the soil environment [16,17,18,19]. Therefore, an in-depth understanding of the formation process of rhizosphere ROS is of great significance for a comprehensive exploration of the biogeochemical processes of rhizosphere soil.

The source of ROS in the rhizosphere involves soil organic matter, plant roots and microorganisms, and root exudates are closely related to plant metabolism, growth and the external environment. At the same time, root exudates affect the species and richness of rhizosphere soil microorganisms. However, there is a lack of comprehensive analysis of the effects of root exudates on the formation mechanism and ecological effects of ROS in the rhizosphere. Therefore, this paper systematically reviews and summarizes the research progress on ROS in rhizosphere soil: (1) the detection method for rhizosphere ROS; (2) the formation mechanism and influencing factors of ROS in rhizosphere soil; (3) the effects of rhizosphere soil ROS on plant growth and health, soil microorganisms, soil organic matter decomposition and nutrient cycling; and (4) the effect of rhizosphere ROS on the degradation and transformation of soil pollutants. Through the above research, it is of great significance to deeply understand the formation mechanism and ecological effect of root exudates on rhizosphere ROS, providing new ideas for revealing the complexity of the soil ecosystem, plant growth and soil ecological environment protection.

## 2. Detection Method for Rhizosphere ROS

ROS have the characteristics of a short life, high reactivity and low concentration in the ecosystem. The complex physiological environment also makes the real-time monitoring of ROS challenging. At present, the analysis and detection methods for ROS mainly include chemiluminescence, fluorescence probe, spectrophotometry and electron paramagnetic resonance spectroscopy [20,21,22,23].

The chemiluminescence method uses the energy released by the molecular reagent reaction to induce the transition of the product molecule, prompting the energy to be emitted in the form of photons and quantitatively detecting ROS by measuring the intensity of the optical signal [24]. There are a few types of probes for the chemiluminescence method, which are mainly divided into lucigenin and luminol [22]. It is generally only used for the detection of O_2_^•−^ and has poor specificity for different types of ROS, so its future development is likely to be limited. The fluorescent probe method is based on ROS’ high oxidation and fluorescent probe reaction. When the fluorescence effect of the product is quite different from that of the original probe, ROS can be quantitatively analyzed indirectly by measuring the fluorescence intensity [25]. Its specificity depends on the type of probe, such as 2′,7′-Dichlorodihydrofluorescein Diacetate (DCFH-DA) for the detection of H_2_O_2_ or Hydroxyphenyl Fluorescein (HPF) for the detection of ^•^OH [26]. This method has a low cost, strong operability, and good biocompatibility. It can evaluate the redox state in organisms and perform real-time in situ analysis of specific ROS. Spectrophotometry is simple to operate and is usually based on specific chromogenic reactions. For example, the nitro blue tetrazolium reduction method is often used to detect O_2_^•−^ [25]. This method realizes semi-quantitative analysis by the difference in absorbance of the product after the reaction of ROS and redox substances at different wavelengths, so it is easy to face problems such as low detection sensitivity and poor selectivity [24]. Electron paramagnetic resonance spectroscopy can achieve the stable capture of free radicals through specific spin traps, which solves the problem of ROS being difficult to directly determine due to the short life, or oxidize them into relatively stable free radicals to be more easily captured by electron spin resonators [27]. This method can distinguish different free radicals and add spin-trapping agents to enhance the specificity, such as DMPO (5-tert-butoxycarbonyl-5-methyl-1-pyrroline N-oxide) [28]. However, this method is easily affected by external conditions such as the pH, temperature and solvent. The analysis method is complex and the equipment cost is high, so it is more suitable for detection applications in non-biological systems.

In summary, electron paramagnetic resonance spectroscopy and fluorescent probe methods are currently highly specific detection methods, which need to combine specific reagents or probes to enhance the selectivity of the target ROS. A summary of the commonly used methods is shown in Table 1.

## 3. Mechanisms of ROS Formation in Rhizosphere

Rhizosphere ROS production is mainly affected by biotic (microbial) and abiotic factors (root exudates, water-soluble phenols and Fe(II)) [37,38,39]. Microorganisms such as bacteria and fungi in the soil are key factors regulating the production of ROS in the rhizosphere. For example, Bacteroidota and Actinobacterta in the bacterial community, both of which belong to the dominant rhizosphere flora, can promote the production of O_2_^•−^ [40,41]. Basidiomycota in the fungal community can trigger extracellular Fenton chemical reactions, thereby promoting the production of ROS in the rhizosphere [42]. Abiotic factors Fe(II) and water-soluble phenols are redox components, which can promote the generation of ROS in the rhizosphere through redox reactions (such as Fe^2+^ + H_2_O_2_ → Fe^3+^ + ^•^OH + OH^−^, Fe^2+^ + Catechol + O_2_ → Fe^3+^ + Quinone + O_2_^•−^ + H_2_O_2_ + ^•^OH) [39]. Root exudates are the medium for material exchange between plant roots and the surrounding environment, and they can promote the interaction between plants and soil microorganisms, which plays an important role in the rhizosphere microenvironment. Root exudates mainly affect the generation of ROS in the following ways. Enzymes (such as peroxidase) in root exudates can directly participate in the generation of ROS [43]. At the same time, organic acids and phenols in root exudates can also directly bind to metal ions to generate ROS [44]. The organic matter in the root exudates can provide carbon and energy sources for soil microorganisms, promote the growth and metabolic activities of microorganisms, and indirectly promote the production of ROS [45].

**Figure 1:**
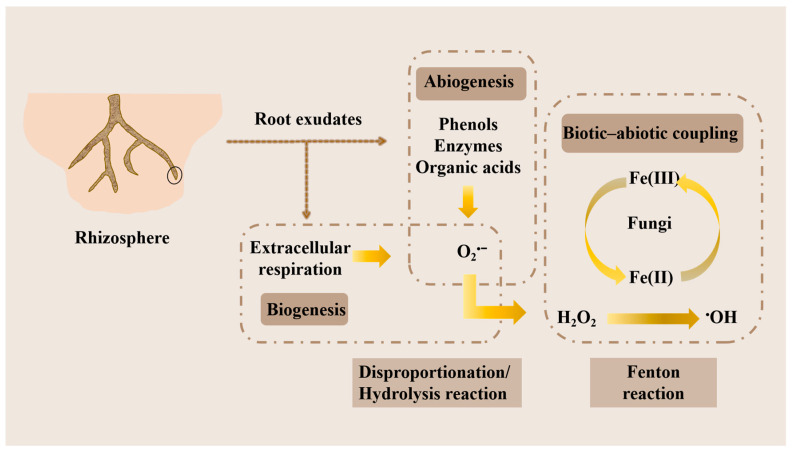
The formation of root-associated ROS is co–regulated by biological and abiotic processes. Biological processes: rhizosphere microorganisms generate O_2_^•−^ through extracellular electron transfer and drive iron cycling, indirectly promoting ROS production. Abiotic processes: water-soluble phenolics/organic acids chemically reduce Fe(III) to Fe(II), which catalyzes H_2_O_2_ decomposition via the Fenton reaction. Microbial-secreted phenolics form redox cycles with Fe(II)/Fe(III), exhibiting dual biological–abiotic roles in ROS generation.

There are many explanations for the formation of rhizosphere ROS in soil. Initially, this process was only attributed to the individual effects of biotic or abiotic factors [38,46], but the soil is a complex structure, and the formation of rhizosphere ROS is not controlled by a single factor. The formation of ROS in the rhizosphere mainly involves the following processes. Firstly, the root exudates secreted by plants during growth can promote the growth of microorganisms and then promote the extracellular release of O_2_^•−^ to enhance the biosynthesis of O_2_^•−^ [38,42]. At the same time, water-soluble phenols can promote the non-biological production of O_2_^•−^. As the main component of root exudates, they have strong redox properties and can promote the transfer of electrons to molecular oxygen [47]. The generated O_2_^•−^ is very unstable, which is converted into H_2_O_2_ through disproportionation (2O_2_^•−^ + 2H^+^ → H_2_O_2_ + O_2_) and hydrolysis reactions (O_2_^•−^ + H_2_O → HO_2_^•^+OH^−^, 2HO_2_^•^ → H_2_O_2_ + O_2_), while H_2_O_2_ can be decomposed into ^•^OH through the Fe(II)-mediated Fenton reaction [37,38] (Figure 1). These results indicate that abiotic factors are key factors in promoting soil ROS production. In addition, biological factors are also crucial for the production of ROS in the rhizosphere. The fungal community can indirectly promote the production of ^•^OH by affecting the Fe(III)/Fe(II) cycle. Fungi such as *Fusarium oxysporum* and *Fusarium solani* can reduce Fe(III) to Fe(II) [48]. In addition, fungi can participate in the iron cycle by carrying extracellular iron, and the metabolites of fungi can also participate in the Fenton reaction to regulate the iron cycle [49,50] (Figure 1). These studies have shown that the coupling of biotic and abiotic factors plays an important role in the production of ROS in the rhizosphere, including the release of extracellular O_2_^•−^ driven by microbial communities, electron transfer mediated by water-soluble phenols and Fe(II), and the effect of fungi on the Fe(III)/Fe(II) cycle. The coupling of microbial communities and water-soluble phenols plays an important role in the formation of O_2_^•−^ and H_2_O_2_, while abiotic factors (Fe(II)) play a dominant and direct role in the formation of ^•^OH. 

## 4. Influencing Factors of ROS Production in Rhizosphere

The biological factors (fungi, bacteria, etc.) and abiotic factors (root exudates, soluble phenols, Fe(II)) mentioned above are important and direct influencing factors in the formation of rhizosphere ROS. In addition, plant growth and environmental factors also affect the formation of ROS. Plant growth can affect the biological and chemical properties of the rhizosphere soil, resulting in changes in the root–soil interface activity, which in turn affects the production of ROS in the rhizosphere [11,51,52]. Studies have shown that ROS accumulate in rice rhizosphere nutrient solution and soil pore water, which is mainly due to the interaction between oxygen released by roots and electrons released by microbial respiration [15]. In addition, during the growth of maize, the ROS content in the rhizosphere soil of maize was 3–15 times higher than that in the non-rhizosphere soil, and it changed significantly with the growth of maize, while the ROS concentration in the non-rhizosphere soil did not change significantly [29].

Studies have found that a large number of ROS are accumulated in the newly developed roots of maize; that is, during the culture process, ROS hotspots are gradually transferred from seed roots to lateral roots, indicating that root development affects ROS production, and new roots are the main contributors to ROS production [29]. Compared with seed roots, lateral roots are usually shorter and smaller, and their secretory functions are different [53,54], resulting in differences in the content and composition of root exudates, which are key factors in promoting microbial growth and metabolism, affecting microbial diversity [53,55]. This series of changes and differences will indirectly affect the formation of ROS. The content of ROS in the rhizosphere increased first and then decreased with the increase in the temperature, and it reached a maximum at 25 °C [29]. At this temperature, the content of root exudates also reached a maximum. Previous studies have found that the photosynthesis and transpiration of plants significantly affect the release of root exudates [55], so the effect of temperature on ROS production in the rhizosphere may be mediated by changing the biochemical properties of the rhizosphere. In addition, considering that an appropriate ambient temperature will promote ROS production, and ROS will participate in mediating biogeochemical processes [29]. Then, climate warming will affect the soil environment, microorganisms, and plant rhizosphere physiological activities, which may stimulate the production of rhizosphere ROS and further lead to the production of greenhouse gases, thereby exacerbating climate change, so this needs our attention. Similar to the temperature, the soil moisture also affects the release of root exudates and indirectly affects the production of ROS, which is closely related to the biochemical properties of the rhizosphere [55,56]. When the soil moisture reached 45% of the maximum field capacity, the content of ROS in the rhizosphere reached a maximum. Other studies have shown that the temperature and soil moisture may also regulate the concentration of water-soluble phenols to mediate ROS production [57,58]. The availability of oxygen is also a non-negligible factor, because oxygen can act as a precursor of ROS and directly promote their production [15], and the content of ROS increases with the increase of oxygen availability.

ROS play a dual role in plant physiological and ecological processes, and the dynamic balance between their production and clearance is significantly regulated by the species characteristics, developmental stages and ages. Plant species diversity affects ROS levels through differences in the metabolic pathways and microbial interactions. Drought-tolerant species such as *Tamarix* spp. have evolved efficient antioxidant systems of superoxide dismutase (SOD) and catalase (CAT), which can quickly remove ROS to avoid oxidative damage [59]. Sensitive species such as Arabidopsis mutants are more likely to accumulate ROS and induce apoptosis due to antioxidant enzyme defects [6]. In addition, the root exudates of different plants significantly affected the rhizosphere microbial community and indirectly regulated ROS production. Root exudates of Gramineae plants such as rice promoted the proliferation of H_2_O_2_-producing Pseudomonas, thereby increasing the level of ROS in the rhizosphere [60].

The developmental stage of plants directly regulates the ROS dynamics. Photosynthesis and respiration are vigorous at the seedling stage, but the antioxidant system is not yet mature, resulting in a short accumulation of ROS. For example, maize seedlings showed increased H_2_O_2_ levels during the light adaptation period, which promoted cell wall relaxation to support rapid growth [61]. The activity of antioxidant enzymes in plants reached a peak at the mature stage, and ROS maintained a steady state. Wheat flag leaf has the highest CAT activity at the heading stage, which can effectively remove H_2_O_2_ produced by photorespiration [62]. The increase in the plant age is accompanied by the decline of redox homeostasis. The electron leakage of mitochondrial complexes I and III in aged plants increased, and the generation rate of O_2_^•−^ increased significantly. The mitochondrial ROS levels in senescent Arabidopsis leaves are up to three times higher than those in young leaves [63]. At the same time, the content of antioxidants such as ascorbic acid (AsA) and glutathione (GSH) decreased with age, weakening the ability of ROS scavenging. The content of AsA in old leaves is only 40% of that in new leaves, resulting in the accumulation of H_2_O_2_ [64]. Epigenetic regulation is also involved in age-related ROS dynamics, such as hypermethylation of the CAT1 promoter region in aging poplar inhibiting its expression and reducing enzyme activity [65]. These mechanisms together lead to decreased ROS scavenging efficiency and aggravated oxidative damage in aged plants.

## 5. Ecological Effects of Root Exudate-Mediated ROS

### 5.1. Effects on Plant Growth and Health

The effects of rhizosphere ROS on plant health are complex, both positive and negative. When plants are infected by pathogens, ROS can rapidly accumulate near the cell wall of plants, and they can also directly attack the cell membranes of pathogens and increase their permeability, leading to the leakage of cell contents, thereby inhibiting the growth and reproduction of pathogens. ROS can also act as a signal molecules to activate defense genes in plants. For example, ROS can induce plants to produce phytoalexins and pathogenesis-related proteins, which can enhance plant disease resistance [66]. An appropriate number of ROS helps plants to establish symbiotic relationships with some beneficial microorganisms. For example, during the symbiosis of leguminous plants and rhizobia, some components of root exudates stimulate rhizobia to produce signaling molecules, which in turn induces ROS in plant roots. In active growth sites such as root tips, an appropriate number of ROS can provide a suitable environment for cell elongation by regulating the relaxation and remodeling of cell walls. At the same time, ROS can also affect the expression of cell-cycle-related genes and promote cell division, thereby promoting the growth of plant roots and aboveground parts [67]. In addition, ROS can also participate in the signal transduction pathways of hormones such as ethylene and abscisic acid, play an important role in physiological processes such as seed germination, leaf senescence, and stomatal movement, and maintain the normal growth and development of plants [68]. However, excessive ROS can lead to oxidative stress in plant cells, attacking intracellular biological macromolecules such as lipids, proteins and nucleic acids. This kind of oxidative damage will destroy the structure and function of the cell membrane, lead to lipid peroxidation of the cell membrane, affect the ion balance and metabolic process in cells, and affect the physiological function and genetic stability of plants [66].

The function of ROS in plant cells is related to the concentration, and a high concentration of ROS may be fatal to the integrity of plant cells. At lower concentrations, ROS play a role in signaling pathways that regulate plant development in response to physiological and environmental stresses [1]. The production of a series of ROS is a by-product of plant metabolic processes, such as *Triticum turgidum* and *Arabidopsis thaliana*. The ROS levels must be maintained at a steady state to ensure proper mitotic microtubule system function. When the concentration of ROS is elevated or insufficient, it will lead to a variety of harmful phenotypes, such as the disappearance of microtubules, the inhibition of early band formation, and the delay of midterm nuclear membrane rupture [69]. Changes in the ROS in the aerenchyma of wheat germ roots during waterlogging were observed, and the subcellular localization of H_2_O_2_ was studied: H_2_O_2_ accumulation was not detected in root cells under aerobic conditions. When the waterlogging experiment was carried out for 6 h, 12 h and 24 h, the accumulation of H_2_O_2_ was detected in the plasma membrane, the inner surface of the plasma membrane and the mitochondrial membrane of the corneal cells. The accumulation of H_2_O_2_ in the cytoplasm was weak, and it could also be detected in the degradation vesicles and cells. After 48 h, the cell contents were basically degraded, and the accumulation of H_2_O_2_ was only found in some degraded vesicles. The above results indicate that during waterlogging, genes regulating ROS homeostasis are differently regulated in wheat roots. ROS induces a fine-grained regulatory mechanism composed of genes encoding ROS-producing enzymes (NADPH oxidase and superoxide dismutase (SOD))/scavenging enzymes (catalase (CAT) and metallothionein (MT)) to strictly control the ROS levels and participate in the cortical cell death process, namely aerenchyma formation [70]. It was observed that the expression of NADPH oxidase was up-regulated and the expression of MT was down-regulated in maize cortical cells. The researchers speculated that the different expressions of these genes controlling ROS homeostasis led to the accumulation of ROS in maize root cortical cells, which triggered the process of programmed cell death (PCD) [71].

ROS can also induce a plant hypersensitive response (HR) to biotrophic pathogens, limiting the spread of pathogens by triggering PCD at the site of infection, which is a non-specific strategy to improve the plant defense [72,73]. Plants selectively limit the proliferation of specific plant microbiome members through the ROS mechanism. Extracellular ROS in the rice rhizosphere significantly affected the rhizosphere microbial community by inhibiting *Geobacter, WPS1, Methylocaldum,* and *Prostocomicrobium* and promoting *Azospirillum*, *Tumebacillus*, *Geothrix* and *Desulfovibrio* [15]. After plants are infected by pathogens, beneficial microorganisms around the rhizosphere are recruited to accumulate in the rhizosphere and change the structure of the microbiome to enhance the resistance to diseases [74,75]. Specific microbiota can reduce toxic ROS levels. It has been reported that *Piriformospora indica* can induce the potential of monocotyledonous plant barley to improve resistance to fungal diseases and tolerance to salt stress. Root endophytic fungi can induce systemic resistance, and this systemic change in the ‘defense preparedness’ state is related to the enhanced antioxidant capacity caused by the activation of the glutathione–ascorbic-acid cycle. It is inferred that higher antioxidant levels protect barley roots from cell death caused by the root pathogens *Fusarium culmorum* and *Cochliobolu sativus*. This is because the production of ROS and the killing of host cells are prerequisites for successful fungal development and necrotrophic pathogenesis [76]. Studies have identified the *Arabidopsis thaliana* FERONIA (FER) receptor kinase mutant FER-8, and its rhizosphere microbiome is rich in *Pseudomonas fluorescens*. FER-8 reduces the basic level of ROS in roots. On the contrary, NADPH oxidase-deficient mutants have increased the number of rhizosphere *Pseudomonas*. Further attempts were made to add the FER ligand–RALF23 peptide, fully demonstrating that FER-mediated ROS production regulates the level of beneficial *Pseudomonas* in the rhizosphere microbiome [77]. Some studies have used cucumbers inoculated with cucumber-specific *Fusarium oxysporum* for eight generations in a split-root system to regulate the soil. It was found that after pathogen infection, the incidence of disease gradually decreased, and the number of ROS (mainly ^•^OH) in the roots increased. *Bacillus* and *Sphingomonas* also accumulated. The above content reveals that the cucumber rhizosphere releases specific compounds (threonic acid and lysine) to enrich beneficial microorganisms, which prevent pathogen invasion by increasing the host’s ROS level [78]. Studies have shown that *Bacillus* has higher tolerance to ROS than *Fusarium oxysporum* [79], and *Sphingomonas* LK11 can produce significant levels of enzyme antioxidants to fight against ROS [80].

Many experiments have proved that a moderate ROS level can be used as a signal molecule to participate in the regulation of plant growth and development, such as promoting root growth and inducing a defense response. However, excessive ROS can lead to oxidative stress, causing damage to cells and inhibiting plant growth. Root exudates significantly change the community structure and function of rhizosphere microorganisms by maintaining the dynamic balance of ROS, and they create a suitable growth environment for plants. After being infected by pathogens, plants recruit beneficial microorganisms around the rhizosphere to enhance their resistance to diseases, and microorganisms can reduce the level of toxic ROS and improve the adaptability of plants to adversity.

### 5.2. Effects on Soil Nutrient Cycling

**Figure 2:**
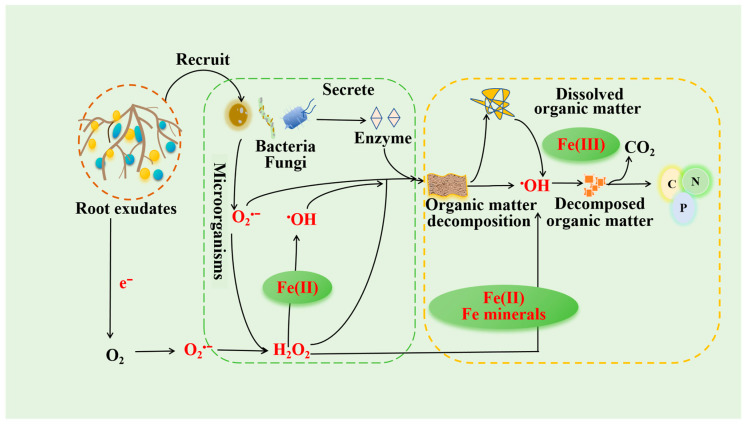
The role of root exudate-mediated ROS in nutrient cycling. Root exudates drive the production of ROS through biological (stimulate microbial metabolism and generate ROS to oxidize organic matter) and non-biological processes (organic acids dissolve iron-bearing oxides, releasing Fe^3+^/Fe^2+^, and react with H_2_O_2_), synergistically promoting organic matter decomposition and nutrient cycling.

ROS play an important role in soil nutrient cycling. Especially under the influence of root exudates, they not only affect the production and distribution of ROS but also some components may act as electron donors or receptors to participate in redox reactions in the soil, thereby inducing ROS formation and promoting soil organic matter (OM) decomposition and nutrient cycling [15,29,81]. Most of the decomposition and mineralization of OM in soil are carried out under the action of microorganisms and their enzymes. In the process of decomposition and mineralization, ROS may be produced as an intermediate product or reaction medium. A variety of ROS are highly reactive, which can further promote the oxidative decomposition of OM and produce CO_2_, thus accelerating the process of soil nutrient cycling [82,83] (Figure 2). At the same time, solid OM plays an important role in the process of activating molecular oxygen to produce ^•^OH. When O_2_ is reduced by reducing the components at the underground aerobic–anaerobic interface, it can generate highly reactive ^•^OH. Fe(II) and reduced OM are considered to be the main reducing components that activate O_2_ to produce ^•^OH in soil/sediment–pore-water environments [84]. In addition, the dynamic distribution of ROS in the detritusphere and the effect on organic carbon (OC) mineralization in soil hotspots were studied [11], and it was found that the detritusphere was a hotspot for ROS production. Among these ROS, the production of O_2_^•−^ and H_2_O_2_ is related to phenol oxidase, water-soluble phenols, and microbial abundance. With the decomposition of residues, the main driving factor in ^•^OH production changed from Fe(II) to water extractable organic carbon (WEOC). Regardless of how the main driving factors change, microbial activity plays a key role in the production of ^•^OH, which indicates the dynamic and complex process of ^•^OH production. The generated ROS had a significant effect on organic carbon mineralization, and different types of ROS contributed differently. ^•^OH and O_2_^•−^ increased the OC mineralization by 15% and 4%, respectively, while H_2_O_2_ reduced it by 18% [83]. The inhibition of organic carbon mineralization by H_2_O_2_ may be due to its strong oxidizability.

Soil OM decomposition and mineralization are also related to abiotic processes. The soil contains a large amount of clay and Fe(oxyhydr) oxides, which can be used as catalysts and nanocatalysts in Fenton or Fenton-like reactions. Fenton oxidation is a catalytic reaction chain between H_2_O_2_, Fe(II) and iron(oxyhydrogen) oxides, which produces highly reactive ^•^OH and oxidizes organic matter to CO_2_ (Figure 2). The direct contribution of the Fenton reaction to OM oxidation and CO_2_ emissions in general soils is less than 0.5%, but in tropical and humid subtropical hot deserts and red soils, this proportion can reach 30% [42]. Non-pyrogenic charcoalification serves as a critical abiotic transformation mechanism, facilitating the conversion of organic carbon into condensed aromatic carbon (ConAC) through iron-mineral-mediated processes coupled with wet–dry cycling or ROS-driven oxidation of dissolved organic carbon (DOC) under ultraviolet radiation [85]. The above shows that the ROS in soil promote the decomposition and mineralization of soil OM and release more nutrients for plant absorption and utilization.

ROS can change the valence state of elements through a series of redox reactions in the rhizosphere soil, thereby changing the solubility and availability of elements. For example, the Fenton reaction can oxidize easily soluble Fe(II) to more insoluble Fe(III) and generate iron oxides (such as goethite, hematite, etc.) while generating ROS. This process not only reduces the solubility of Fe(II) but also may fix other metal ions or organic substances through the formation of iron oxides [86], which is not conducive to the absorption and utilization of metal ions by plants. An appropriate number of ROS can promote the growth of beneficial microorganisms, such as rhizobia and phosphate-solubilizing bacteria, which play an important role in soil nutrient cycling. Rhizobia can fix nitrogen in the atmosphere and provide nitrogen for plants. Phosphate-solubilizing bacteria can decompose organic phosphorus or insoluble phosphorus minerals and convert them into plant-absorbable forms [87], but when inoculation demonstrates measurable effects, they are often attributed to the release of soil phosphorus, whereas they are more likely driven by pH-mediated enhancement of the plant phosphorus uptake efficiency [88]. Furthermore, regarding the field adaptability of inoculated microorganisms, it lacks robust experimental evidence and needs to be studied further. The release of carboxylates (e.g., citrate and oxalate) by plant roots may serve as an effective strategy to enhance soil phosphorus solubility through the mobilization of humus-associated phosphorus. This is because carboxylates can mobilize not only orthophosphate anions but also highly phosphorylated phytic acid (inositol hexakisphosphate). Given that highly phosphorylated phytic acid exhibits strong binding to soil solid phases (e.g., Fe/Al oxides and humic complexes), the carboxylate-mediated mobilization process appears critical for plant acquisition of inositol-P, a key organic phosphorus form in soils [89].

The ROS in rhizosphere soil have high chemical activity, which can react with amino acid residues (such as cysteine) and active centers in soil enzyme molecules, change the spatial structure and active sites of enzymes, and thus affect the activity of enzymes. For example, O_2_^•−^ and H_2_O_2_ can oxidize cysteine residues in soil urease, resulting in a decrease in urease activity, which in turn affects urea hydrolysis and nitrogen transformation [90]. ROS can also promote the cross-linking and polymerization of organic substances in soil through oxidation reactions to form macromolecular organic–inorganic complexes, thereby bonding soil particles together and increasing the stability of soil aggregates. This stable soil aggregate can provide a suitable living environment for soil microorganisms, which is conducive to the decomposition of organic matter and nutrient transformation by microorganisms [91].

Through the coupling process of biotic and abiotic redox, the rhizosphere has become a common hotspot for ROS production, participating in the decomposition and transformation of soil organic matter, affecting nitrogen transformation and phosphorus activation, thus changing the effectiveness and circulation efficiency of soil nutrients. In addition, the decomposition and transformation of soil OM affects the release of CO_2_, which in turn has a profound impact on the global carbon cycle and climate change.

### 5.3. Effects on Degradation of Soil Pollutants

**Figure 3:**
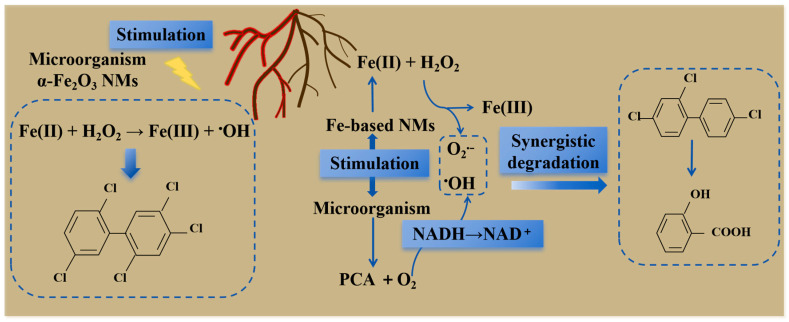
Role of root exudate-mediated ROS in pollutant degradation. The root exudates dissolve Fe-based nanomaterials (NMs) or α-Fe_2_O_3_, releasing Fe^2+^ to trigger the Fenton reaction and generate ^•^OH. Concurrently, the NMs stimulate *Pseudomonas* sp. JD37 to secrete pyocyanin-1-carboxylic acid (PCA), which accelerates NADH/NAD^+^ cycling and promotes O_2_^•−^ production. These ROS degrade 2,4,4′-trichlorobiphenyl (PCB28) into benzoic acid. Furthermore, the synergistic interaction between α-Fe_2_O_3_ NMs and JD37 drives a heterogeneous Fenton reaction. Through Fe^3+^/Fe^2+^ cycling and the formation of iron-oxide–silicate composite films, the system achieves highly efficient degradation of highly chlorinated pollutants.

The extracellular ROS formed by the interaction of rhizosphere microorganisms and plants can promote the removal of soil pollutants. Plant-growth-promoting rhizobacteria (PGPR) isolated from potato rhizosphere soil can produce extracellular ROS to protect plants from external pressure. When Fe-based nanomaterials (20 nm and 100 nm zero-valent iron nanomaterials), α-Fe_2_O_3_ and ferrous ions were treated with *Pseudomonas aeruginosa* (JD37), they could promote the degradation of 2,4,4′-trichlorobiphenyl (PCB28). These nanomaterials stimulated the secretion of pyocyanin-1-carboxylic acid (PCA) by JD37 and accelerated the conversion of NADH/NAD^+^, thereby promoting the production of O_2_^•−^. At the same time, JD37 increased the dissolution of Fe(II) in nano-zero-valent iron and promoted the formation of ^•^OH. These ROS gradually degrade PCB28 into benzoic acid through dihydroxy substitution, oxidation to quinone and the Michael addition reaction (Figure 3). This indicates that iron-based nanomaterials promote ROS production and PGPR can also produce extracellular ROS, which synergistically degrade soil organic pollutants [92]. In addition, the iron plaque mediated by the interaction between α-Fe_2_O_3_ nanomaterials and alfalfa-plant–rhizosphere-bacteria symbionts achieved in situ Fenton oxidation of high-chlorine persistent organic pollutants (2,2′,4,5,5′-pentachlorobiphenyl, PCB101). The co-existence of α-Fe_2_O_3_ nanomaterials and JD37 stimulated the secretion of acidic and reducing substances and H_2_O_2_ in the roots, which jointly mediated the rhizosphere Fenton reaction and converted α-Fe_2_O_3_ nanomaterials into Fe(II)–silicate-rich iron plaque [93] (Figure 3). The residual concentration of polycyclic aromatic hydrocarbons (PAHs) in the rhizosphere soil of maize decreased with the growth of the maize, and the attenuation efficiency of the PAHs showed a similar trend to that of ROS production, which indirectly indicated that ROS in the rhizosphere may play an important role in PAH attenuation. By comparing the differences in the PAH residual concentrations before and after the addition of p-benzoquinone and coumarin, it was found that the contribution rate of the two was between 31.4% and 43.3%. This indicates that ROS-mediated abiotic processes in the rhizosphere play an important role in PAH removal [29]. In addition, the ^•^OH generated during the oxidation of reduced humic acids provides a novel pathway for ROS production under both aerobic and hypoxic conditions. These ROS can initiate the transformation of organic pollutants such as PAHs bound to humin [94]. Four types of natural humic substances (HSs) have been shown to significantly accelerate the photosensitized degradation of bisphenol A (BPA), with ROS produced during HS photochemical reactions serving as the primary driving force for degradation [95]. However, recent studies indicate that humic-substance-mediated ^•^OH can also be generated via dark reactions [96]. These findings highlight the role of extracellular ROS produced by the interaction of rhizosphere microorganisms and rhizosphere plants in the degradation of organic pollutants in dryland soil. At the same time, iron-based nanomaterials play a role in promoting plant–rhizosphere microbial technology to ensure safe crop production and soil remediation, which will help to design new bioremediation technologies for environmental pollution.

The ^•^OH produced during the oxygenation of anoxic paddy mud can non-selectively oxidize most organic substrates, including PAHs [97]. Studies have found that active Fe(II) species, such as exchangeable Fe(II), surface-bound Fe(II) or Fe(II) in low-crystalline minerals, can drive the formation of ^•^OH during redox fluctuations in different paddy soils, which plays a key role in the degradation of organic pollutants, especially degrading PAHs through hydroxylation and ring-opening [97]. Under anoxic conditions, PAHs in paddy mud experienced significant microbial degradation, and the concentrations of naphthalene, phenanthrene and pyrene decreased significantly, but their degradation rates were different when the mud was exposed to oxygen. The degradation products of PAHs were identified, mainly including hydroxylated polycyclic aromatic hydrocarbons (9-phenanthrol), aldehyde products (phenanthrene-4,5-dialdehyde), carboxylated products (2,2′-biphenyldicarboxylic acid) and low-molecular-weight acids (phthalic acid). The study also monitored the dynamic changes in these products. The hydroxylation products of PAHs (1-naphthol, 9-phenanthrol and 4-pyrene phenol) increased rapidly within 2 h and then decreased [97]. These results demonstrate that ^•^OH initiates the oxidation process of PAHs through hydroxyl addition, and the generated products are further oxidized, generating aldehyde products, carboxyl products, low-molecular-weight compounds through ring-cracking and oxygenation mechanisms, eventually generating CO_2_ and H_2_O [98]. When propanil (2 mg/L) was added to the culture medium of rice plants, it was found that the concentration of propanil decreased gradually, which may be due to its adsorption by the iron film, oxidative degradation by ^•^OH or absorption by rice plants. When sodium formate was added, the removal rate of propanil was significantly reduced, indicating that the ^•^OH produced by the iron film led to the degradation of propanil [19]. In soil/sediment, the production of ^•^OH is closely related to the redox conditions, mainly produced by the aqueous phase Fe(II). During the oxidation process, ion-exchanged Fe(II) and surface-adsorbed Fe(II) were rapidly oxidized within 1 h, while the oxidation of mineral-structured Fe(II) was first rapidly oxidized and then slowly oxidized. Highly active mineral-structured Fe(II) is the main contributor to the activation of molecular oxygen to produce ^•^OH.

The synergistic effect of rhizosphere microorganisms and plants significantly promotes the degradation of organic pollutants by producing extracellular ROS. Fe-based nanomaterials promote ROS production and improve pollutant degradation efficiency. There are obvious differences in the degradation of organic pollutants between the maize rhizosphere and paddy soil, which is mainly related to their root structure, soil environment and microbial community composition. The oxidative degradation system driven by ROS in the maize rhizosphere is more advantageous under aerobic conditions, while anaerobic microorganisms in paddy soil also affect the degradation path of pollutants through reduction, which needs to combine the coupling effect of reduction–redox and oxidation.

Heavy metal stress can trigger the accumulation of plant defense mechanisms, such as ROS, plant hormones, NO and other signaling molecules, thereby reducing plant toxicity [99,100]. In order to cope with excessive ROS, the antioxidant defense system of plants is activated. Antioxidant enzymes in plants, as an important part of the defense system, help to eliminate ROS and reduce plant damage [101,102]. Rice produces a micro-oxygen environment in the rhizosphere through radial oxygen secretion, thereby generating an plaque film on the root surface and within a few millimeters of the surrounding area. Iron plaque can adsorb and fix heavy metals such as Cu, Ni, As, and Cd, hindering the absorption of them by plant roots, thereby reducing the accumulation of these elements in rice [103,104,105,106,107]. For example, the application of iron oxide nanoparticles (FeO-NPs) in Cd-contaminated soil reduced the uptake of Cd by wheat by 72.5%. This may be due to the fact that FeO-NPs adsorb and fix Cd through their surface capping molecules, chemical reactivity, electrostatic interaction, and large specific surface area, thereby limiting the migration and bioavailability of Cd. FeO-NPs and Cd^2+^ competed for the same transport channel to enter the plant system, which also reduced the concentration of heavy metal ions in plants. At the same time, the application of FeO-NPs also triggered the production of enzymatic and non-enzymatic antioxidants, thereby scavenging ROS and alleviating oxidative stress in plants. FeO-NPs can also improve soil nutrients (such as N, P and K) [108]. Studies have shown that under Cd stress, FeO-NPs can significantly reduce electrolyte leakage and the malondialdehyde and H_2_O_2_ concentrations, and they can increase the activity of antioxidants in plants [109,110].

The adsorption of Cd by iron plaque was mainly affected by the competition between NH_4_^+^ in soil and the adsorption sites of iron plaque, and the adsorption of As was mainly affected by the radial oxygen loss rate (ROL) and the number of iron plaques. The abundance of ROL and iron-plaque-related microorganisms (OM-degrading bacteria and iron-reducing bacteria (FeRB)) is the main driving factor behind the formation of iron plaque, while iron-oxidizing bacteria (FeOB) in rhizosphere soil have a negative effect [107]. The coexistence of As(III) and As(V) immobilized by iron plaque appeared in the roots of *Typha latifolia*. Compared with pore water, the proportion of As(III) measured in the iron plaque was lower than that of As(V), which may be due to the oxidation of As(III) in the rhizosphere, the change in the As(III)–As(V) ratio in pore water during the formation of iron plaque, or the low adsorption affinity of As(III) [111]. Iron plaque and rice root ROL work together to promote the formation of ^•^OH through the Fenton reaction. Their role in the oxidation and transformation of rhizosphere pollutants has been described previously. These ^•^OH can be used to convert As(III) into As(V) and reduce its toxicity [19], but this needs to be confirmed by research. Iron plaque reduced the concentration of Cr in rice plants by adsorption. A low concentration of Cr(VI) promoted the growth of rice roots, while a high concentration of Cr(VI) reduced the concentration of Fe in rice plants and weakened the formation of iron plaque [112]. There are few studies on how iron plaque effectively fixes Cr and reduces Cr(VI), and this remains to be explored.

## 6. Conclusions and Prospects

Root exudates, serving as a crucial medium for communication between roots and the soil environment, have significantly regulatory effects on the production of ROS in the rhizosphere. Various research methods have demonstrated the presence and accumulation of ROS in the rhizosphere, with the concentration being significantly higher than that in bulk soil. The generation of ROS in the rhizosphere is co-regulated by biotic and abiotic factors, playing a vital role in modulating plant growth, health, and responses to stress, as well as exerting important influences on the soil carbon turnover, nutrient cycling, and pollutant transformation. Therefore, in-depth investigation of the formation mechanisms of rhizosphere ROS and their impacts on the soil environment is of great significance for understanding the interactions between plants and soil, as well as for enhancing soil fertility and environmental quality.

At present, there have been innovative breakthroughs in the detection methods for ROS, offering more options for ROS research. However, the existing research methods have some limitations, and further in-depth studies are required to understand how the coordinated effects of multiple factors influence the production of ROS and the ecological consequences. Future research should focus on the following aspects.

The detection methods for ROS should be improved, such as developing chemical luminescent probes with higher specificity to enhance the ability of chemiluminescence methods to detect multiple types of ROS; optimizing the performance of fluorescent probes to improve their stability and sensitivity; and exploring ways to reduce the equipment costs of electron paramagnetic resonance (EPR) spectroscopy and minimize the influence of external conditions, so that it can be more widely applied in the detection of biological systems.

Conduct an in-depth analysis of the interaction patterns between root exudate components and the ROS generation network. A key focus should be on unraveling the molecular pathways through which characteristic exudates such as organic acids and phenolics influence the Fenton reaction by regulating the iron redox cycle. This should be achieved by combining single-cell sequencing and spatial metabolomics technologies to elucidate the regulatory mechanisms of the spatiotemporal heterogeneity of exudates on the formation of ROS hotspots during different root developmental stages and under stress conditions. Secondly, emphasis should be placed on the synergistic effects of the microbe–plant–mineral ternary system on ROS production, particularly how the interaction between rhizosphere siderophore-producing microorganisms and the plant iron uptake system modulates the intensity of the Fenton reaction by altering the bioavailability of iron.

Construct a multi-factor coupling model to integrate the effects of multiple stress signals, such as the temperature, moisture, and pollutants, on ROS homeostasis. By establishing an in situ ROS visualization monitoring system in the rhizosphere, decipher the dynamic reprogramming process of ROS signaling pathways under environmental gradient changes, providing a theoretical basis for the development of intelligent stress-resistant cultivation techniques based on ROS signaling regulation.

From the perspective of application and transformation, combine research findings with agricultural production practices, strengthen research on rhizosphere ROS-mediated soil processes, and investigate the impact of different agricultural management practices (such as organic fertilizer application and water regulation) on the regulation of ROS by root exudates. Optimize the rhizosphere ROS levels to enhance plant stress resistance and growth capacity. Leverage the relationship between root exudates and ROS to promote the decomposition of soil organic matter and nutrient cycling, as well as accelerate the degradation and transformation of pollutants. Provide new ideas and methods for sustainable agricultural development, soil environmental quality improvement, and the restoration and protection of ecosystems.

## Figures and Tables

**Table 1:** Common methods for detecting rhizosphere ROS.

Methods	Applicable Scenarios	Advantages	Disadvantages	References
Chemiluminescence	Assess the degradation mechanism of soil pollutants	High sensitivity; real-time monitoring of ROS production	Low specificity; vulnerable to environmental factors	[29,30]
Fluorescence probe	Study the interaction between plant roots and microorganisms	Simple operation; low cost; detect ROS at low concentrations	Limitation of the probe stability; easily affected by organic matter or minerals	[31]
Spectrophotometry	Assess the redox status of rhizosphere soil; detect the soil microbial activity	Simple operation; low cost; carry out quantitative determination; wide range of applications	Real-time monitoring cannot be achieved; limited sensitivity	[32]
Electron paramagnetic resonance spectroscopy	Assess the soil redox status; analyze the plant responses to environmental stresses	High specificity; no marking required; suitable for complex environments; real-time monitoring	High cost of instruments; high requirements for the uniformity and stability of samples	[33,34]
High-performance liquid chromatography	Detect the oxidation products in rhizosphere soil; evaluate the metabolic activity of soil microorganisms	High sensitivity and high accuracy; complete the analysis in a short time; wide applicability	High cost of instruments; complex operation; not suitable for large-scale sample detection	[35]
In vivo imaging technology	Study the temporal and spatial variation of ROS in the rhizosphere	Real time and dynamic; high resolution	High cost of instruments; complex operation	[29]
Iodine reduction titration method	Evaluate redox changes during soil pollution remediation	Simple operation; low cost; wide range of application; fast detection speed	Cannot distinguish different types of ROS; unable to perform real-time monitoring; high reaction conditions	[36]

## Data Availability

All data are included in the main text.
